# Its Work and Development

**Published:** 1918-09-21

**Authors:** 


### THE METROPOLITAN ASYLUMS BOARD IN 1917.

### Its Work and Development.

In pre-war days the annual report of the Metropolitan Asylums Board was always full of interest, with elaborate statistical tables and a fully detailed account of the Board's work. For the past three years the claims of the war have made it necessary to curtail these reports, and that for 1917 is merely a brief summary of what has been done during the year. It shows, however, how greatly the duties of the Board have been increased since it was formed and how important a factor it is now in the Public Health work of London. An Order of the Poor-Law Board dated May 15, 1867, established the Board "to receive and relieve poor persons, chargeable to some union or parish in the Metropolitan area, suffering from fever, small-pox, or insanity." A further Order in 1875 defined the insane who were to be received as "such harmless persons of the chronic or imbecile class as could be lawfully retained in a workhouse." In the same year the Local Government Board sanctioned the provision of a ship for preparing Poor-Law boys for sea service. In 1888 Parliament gave the Board powers to admit any person suffering from fever or small-pox whether chargeable to the Poor Law or not, and extended these powers to cases of diphtheria.

Since these early years the work of the Board has increased continuously. In 1897 Orders were made for the provision of accommodation for Poor-Law children suffering from ophthalmia, ringworm, or tubercular disease, or requiring sea-side air or special treatment in a hospital or convalescent home, and for feeble-minded Poor-Law children not under certificates; in 1905 for cases of plague or cholera; in 1907 for cases of cerebro-spinal meningitis; in 1910 for Poor-Law patients suffering from measles—extended to all patients in 1912; in 1911 for Poor-Law patients suffering from whooping-cough—extended to all patients in 1912; in 1911 the Board also took over the charge of the casual poor; in 1912 the care of cases of puerperal fever was added to the Board's duties; in 1913 tubercular cases received from the London Insurance Committee and extra-Metropolitan County Councils; in 1916 sane epileptics and parturient women, chargeable to the Poor Law, suffering from venereal disease; and in 1917 cases certified under the Mental Deficiency Act of 1913, cases of ophthalmia neonatorum and discharged soldiers suffering from advanced tuberculosis. Add to this that the Board provides an Ambulance Service and central stores for its numerous institutions, conducts bacteriological and research work in connection with the diseases it treats, collects and distributes information on the incidence of infectious disease, provides instruction in fever work to medical men, trains nurses in fever nursing, and since the outbreak of the war has undertaken the care of a large number of Belgian refugees, and a clear idea can be formed of the multifarious duties for which the Board is responsible.

The work is now being carried on under great difficulties. Speaking of the staff and of military service the report says plaintively, in a phrase the meaning of which is clear though the wording is quaint, "the conditions are now such that practically nothing is left to the discretion of the Board except to comply with the requirements of the Government in the matter." The difficulty the Board has had in getting and keeping a staff is shown by the fact that at the end of the year there were about 600 vacancies. In this lies the justification for the Board's recent action in asking medical men not to send slight cases of scarlet fever and diphtheria to its hospitals.

The special war-work of the Board has consisted in handing over four of its hospitals to the Army Medical Service, and in providing accommodation for Belgian refugees. The number of Belgians provided for has naturally fallen off very considerably. In 1917, 7,218 were admitted, as compared with 12,311 in 1916, 58,474 in 1915, and 51,769 in 1914.

### No Case of Small-Pox.

The total number of persons admitted to the institutions of the Board in 1917 was 35,808—1,202 fewer than those admitted in 1916. Most of the admissions were cases of infectious diseases, the number being 20,246, 4,471 less than in 1916. The tables show that scarlet fever and diphtheria are the diseases most readily sent into the hospitals. Of 6,137 cases of scarlet fever notified, 5,294 were admitted; of 8,298 cases of diphtheria, 7,391; of 457 cases of enteric fever, 172; of 199 cases of puerperal fever, 61; and of 405 cases of cerebro-spinal fever, 101. The number of cases of measles treated was 1,009, as compared with 470 in 1916. No case of small-pox was treated during the year, three cases were notified, but the diagnosis was not confirmed. During the year the Board arranged with the Guardians of the City of London to receive into their institution at Thavies Inn parturient women suffering from venereal disease. The first admission was on September 7, and by the end of the year thirteen cases had been received.

The decrease in the numbers of casual poor which began when the Board took over the charge of the casual wards from the London Boards of Guardians continues. In 1917 the number of casuals received was 8,844, in 1916 it was 9,865.

The usual routine work in the bacteriological laboratories was carried out, but the research work ceased owing to the fact that Dr. Mair, the Board's research pathologist, was called up for military service in June. Shortage of paper, no doubt, accounts for the fact that there are no reports on the clinical work of the hospitals from the medical staff. It is much to be regretted, however, that time and space have not been found for the presentation of statistics showing the after-results of the treatment of phthisis at the Board's sanatoria.

